# Update: Greenland and Robins (1986). Identifiability, exchangeability and epidemiological confounding

**DOI:** 10.1186/1742-5573-6-3

**Published:** 2009-09-04

**Authors:** George Maldonado

**Affiliations:** 1 University of Minnesota School of Public Health, Minneapolis, USA

## Abstract

We are pleased to publish an update to "Identifiabiliity, exchangeability and epidemiological confounding" (IEEC) by Sander Greenland and James Robins, originally published in 1986 in the International Journal of Epidemiology. This is the first in a series of updates to classic epidemiologic-methods papers that EP&I has commissioned.

When EP&I was launched, we planned to commission updates to classic methods papers. We thought it would be interesting and useful to see what changes the original authors would make to a must-read paper if that paper were written today. We are pleased to publish the first paper in this series: an update to "Identifiabiliity, exchangeability and epidemiological confounding" (IEEC) by Sander Greenland and James Robins [1], originally published in 1986 in the International Journal of Epidemiology [2].

IEEC is widely considered to be one of the most important methods papers ever to be published in the epidemiological literature. In the original paper, Greenland and Robins focused on the situation in which the exposed group is the target population and the disease-frequency measure of interest is the incidence proportion, and did the following:

• They explained why the causal effect of an exposure (i.e., a causal contrast) cannot be estimated from data without making assumptions.

• They described these assumptions.

• They drew a logical connection between non-identifiability of a causal contrast and the concept of confounding (and in doing so gave a precise definition of confounding).

• They used these connections to explain the sense in which random allocation to exposure status "controls" confounding.

• They critically evaluated the standard "properties" of a confounder, and showed them to be imperfect.

For me, the above messages were (and continue to be) important. But I also found the original paper to be important for three additional reasons. It caused me to understand the concept of confounding in its fundamental essence (not in terms of covariate control). It helped me to understand counterfactual reasoning (even though they did not use that term) and its utility for thinking about etiologic studies. And it introduced me to a simple deterministic model of effects (what readers may recall as the p's and q's model) that I find to be a very powerful tool for thinking about epidemiologic methods.

Many thanks to Greenland and Robins for this update.
